# Bisphosphonate-Based Molecules as Potential New Antiparasitic Drugs

**DOI:** 10.3390/molecules25112602

**Published:** 2020-06-03

**Authors:** Joice Castelo Branco Santos, Jonathas Alves de Melo, Sweta Maheshwari, Wendy Marina Toscano Queiroz de Medeiros, Johny Wysllas de Freitas Oliveira, Cláudia Jassica Moreno, L. Mario Amzel, Sandra B. Gabelli, Marcelo Sousa Silva

**Affiliations:** 1 Immunoparasitology Laboratory, Department of Clinical and Toxicological Analysis, Health Sciences Center, Federal University of Rio Grande do Norte, 59012-570 Natal, Brazil; joic.cast@hotmail.com (J.C.B.S.); biomedjonathas@outlook.com (J.A.d.M.); wendymmedeiros@gmail.com (W.M.T.Q.d.M.); johny3355@hotmail.com (J.W.d.F.O.); claudia.mrn1@gmail.com (C.J.M.); 2 Postgraduate Program in Pharmaceutical Sciences, Health Sciences Center, Federal University of Rio Grande do Norte, 59012-570 Natal, Brazil; 3 Postgraduate Program in Biochemistry, Biosciences Center, Federal University of Rio Grande do Norte, 59012-570 Natal, Brazil; 4 Department of Biophysics and Biophysical Chemistry, Johns Hopkins University School of Medicine, Baltimore, MD 21205, USA; sweta@jhmi.edu (S.M.); mamzel@jhmi.edu (L.M.A.); 5 Department of Medicine and Oncology, Johns Hopkins University School of Medicine, Baltimore, MD 21205, USA; 6 Global Health and Tropical Medicine, Institute of Hygiene and Tropical Medicine, New University of Lisbon, 1800-166 Lisbon, Portugal

**Keywords:** bisphosphonate, farnesyl pyrosphosphate synthase, leishmaniasis, chagas disease, neglected tropical diseases

## Abstract

Neglected tropical diseases such as Chagas disease and leishmaniasis affect millions of people around the world. Both diseases affect various parts of the globe and drugs traditionally used in therapy against these diseases have limitations, especially with regard to low efficacy and high toxicity. In this context, the class of bisphosphonate-based compounds has made significant advances regarding the chemical synthesis process as well as the pharmacological properties attributed to these compounds. Among this spectrum of pharmacological activity, bisphosphonate compounds with antiparasitic activity stand out, especially in the treatment of Chagas disease and leishmaniasis caused by *Trypanosoma cruzi* and *Leishmania* spp., respectively. Some bisphosphonate compounds can inhibit the mevalonate pathway, an essential metabolic pathway, by interfering with the synthesis of ergosterol, a sterol responsible for the growth and viability of these parasites. Therefore, this review aims to present the information about the importance of these compounds as antiparasitic agents and as potential new drugs to treat Chagas disease and leishmaniasis.

## 1. Introduction

Neglected tropical diseases (NTDs) are responsible for serious public health problems in much of the world, particularly in developing countries located in Latin America, Africa, and Asia, and can also affect non-endemic developed regions, such as North America and Europe, owing to population migration and climate change. According to the World Health Organization (WHO), more than one billion people are affected by one or more NTDs in 149 countries [1]. Among such diseases are Chagas disease and leishmaniasis, which are caused by protozoa of the order *Kinetoplastida* and family *Trypanosomatidae* [2]. Currently, the therapeutic regimens used to control the foremost NTDs have several limitations, especially concerning pharmacological efficacy, toxicity, high costs, and complicated clinical administration. Furthermore, as these are diseases mostly related to poverty in developing countries, investment in measures to control these diseases are still insufficient [2,3]. Drugs currently used to treat Chagas disease and leishmaniasis caused by *Trypanosoma cruzi* and *Leishmania* spp., respectively, have among their disadvantages the long-term parenteral administration, development of resistance, and the requirement of complex therapeutic schemes [4,5]. Additionally, the high cost and low pharmacological efficacy justify the search for new antiparasitic drugs [6].

In recent years, several strategies have been reported aiming at the development of new drugs with antiparasitic activity, mainly in the context of infections caused by trypanosomatids, such as *Leishmania* spp [7,8,9,10] and *T. cruzi* [11,12,13]. In this scenario, compounds based on bisphosphonates have been gaining prominence owing to their reported antiparasitic activity [7,8,9,10,11,12,13]. Moreover, some of these compounds are already approved for use in humans for the treatment of some bone diseases [14,15]. Bisphosphonates are known to act on the classical mevalonate (MVA) pathway, which is responsible for the synthesis of essential isoprenoids in eukaryotes, archaea, and some bacterial species [16,17]. Specifically, they inhibit the farnesyl pyrophosphate synthase (FPPS), the branch point and [18] rate-limiting enzyme of the MVA pathway [19,20,21].

FPPS has been shown to be essential for the survival of trypanosomatid parasites—*Leishmania major* promastigotes as well as amastigotes and *T. brucei* [8,22]. Furthermore, FPPS can be potently inhibited by bisphosphonates in human pathogens—*Toxoplasma gondii*, *Plasmodium falciparum*, *T. cruzi*, *T. brucei*, *L. major,* and *L. donovani* [23,24,25,26,27,28,29,30,31,32,33]. Taken together, these findings validate FPPS as a target for drug development using bisphosphonates in the treatment of leishmaniasis as well as trypanosomiasis. Thus, the objective of this work is to present the pharmacological applications of bisphosphonates as potential antiparasitic drugs with an emphasis on the therapeutic control of Chagas disease and leishmaniasis.

## 2. History of the Use of Bisphosphonates

The synthesis of organic compounds containing phosphorous was first reported in 1820 by Jean Louis Lassaigne. At that time, alkylphosphonates were synthesized by the condensation of alcohols and phosphoric acids [34]. Later, several compounds were produced using phosphorus as the central atom, called organophosphors, such as triphosphate nucleosides and perfluoroalkylated phosphines [35]. One of the organophosphorus classes is bisphosphonates, which were initially synthesized in 1865 in Germany and have been studied extensively since the 1960s [36].

The knowledge about its use came to light owing to studies on inorganic pyrophosphate, a precursor to bisphosphonates, wherein it was observed that plasma and urine contained compounds that inhibited the precipitation of calcium phosphate and that part of this inhibitory activity was the result of inorganic pyrophosphate [15,37]. This discovery became interesting for pharmacological applications in the treatment of clinical disorders caused by the bone resorption mechanism, such as Paget’s disease, osteoporosis, hypercalcemia, and fibrous dysplasia. However, pyrophosphate is metabolically unstable because it is rapidly hydrolyzed in the gastrointestinal tract. Thus, more stable compounds were sought, such as bisphosphonates [34,35].

Bisphosphonates were initially used by the chemical industry, mainly as corrosion inhibitors or as complexing agents in the textile, fertilizer, and oil industries, in addition to preventing flaking thanks to their ability to inhibit calcium carbonate precipitation [38]. Only in the last three decades have bisphosphonates been developed as drugs for use in various diseases of bone, dental, and calcium metabolism [39]. In this context, etidronate was the first bisphosphonate to be used pharmacologically in patients with ossifying myositis, a heterotopic ossification characterized by the occurrence of bone formation in soft tissues, usually muscle tissue [38,39].

## 3. Chemical and Biological Characteristics of Bisphosphonate-Based Compounds

**Figure 1:**
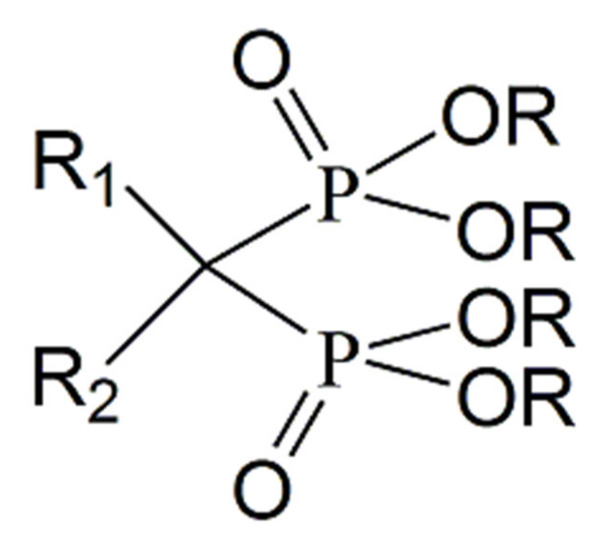
Basic chemical structure of bisphosphonates. The phosphate-carbon-phosphate (P-C-P) bond is the base skeleton of the structure with two covalent side chains (R1 and R2) attached to the germinal carbon. The group R1 allows higher affinity to hydroxyapatite and the group R2 increases the potency of the anti-resorptive capacity and mimic structures that give it different mechanisms of action, whether as adenosine triphosphate (ATP) analogues or isoprenoid pyrophosphate [41].

Chemically, bisphosphonates are classified as a class of metabolically stable pyrophosphate analogue compounds in which the oxygen atom between the two phosphorus atoms in the pyrophosphate is replaced by a carbon atom (P-C-P bond), making these compounds resistant to chemical and enzymatic hydrolysis [8,40]. These compounds have the general structural formula presented in Figure 1. Additionally, bisphosphonates have two substituents in their structure, R1 and R2, linked to the geminal carbon, which allows the versatility of synthesis of this class of compounds with different pharmacological applications [41].

It is observed that, when R1 is a hydroxyl, this group, together with the phosphate groups, facilitates the association with calcium, which ensures strong interaction with bone structures. On the other hand, the R2 group is responsible for determining the bone anti-resorptive power and, depending on the class, their mechanism of action varies. Additionally, groups R1 and R2 can be extensively modified by the chemical synthesis process, enabling new derivatives for this class of molecules, consequently potentiating a diversity of biological activity [40,41,42,43].

Bisphosphonate-based compounds have essential and relevant pharmacological applications. Similar to pyrophosphate, bisphosphonates exhibit a high affinity for bone hydroxyapatite and effectively prevent calcification [44]. The Food and Drug Administration (FDA) currently approves some of these compounds for the treatment of bone resorption, Paget’s disease, osteoporosis, multiple myeloma, hypercalcemia associated with bone metastasis, and fibrous dysplasia [14,15,45]. On the other hand, in addition to their capacity as inhibitors of bone resorption, bisphosphonates are antimicrobial agents [46], anticancer agents [47], selective inhibitors of acid sphingomyelinase, and stimulators of γδ-T lymphocytes [48].

The antiparasitic activity of some bisphosphonates has been shown to be owing to their selective inhibitory capacity in the biosynthesis of isoprenoids [15], including in the context of infections caused by protozoa of medical importance, such as *Toxoplasma gondii* [12,25], *Plasmodium falciparum*, *Trypanosoma cruzi* [11,12,13], *Trypanosoma brucei* [49,50], and *Leishmania* spp. [7,8,9,25], the etiologic agents of toxoplasmosis, malaria, Chagas disease, African sleeping sickness, and leishmaniasis, respectively.

**Figure 2:**
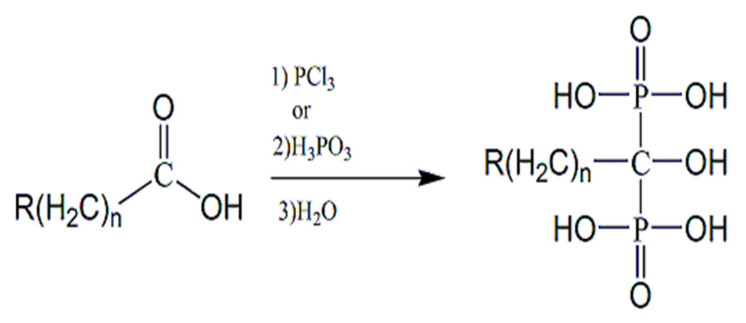
Classical route for the synthesis of hydroxy-bisphosphonates. The method requires the addition of (1) phosphorus trichloride or (2) phosphorous acid to that of carboxylic acid, followed by (3) hydrolysis to generate the bisphosphonate.

As bisphosphonates are a diversified class of chemical compounds, several methods of chemical synthesis have already been described, methods ranging from thermal dehydration at a high temperature to Michael’s reaction [51,52]. The most used method to obtain bisphosphonates is the synthesis from carboxylic acids and their derivatives [53,54]. This method consists of a reaction between a carboxylic acid and a mixture of phosphorus trichloride or phosphorous acid, followed by hydrolysis under acidic conditions to produce hydroxy-bisphosphonates or their sodium salts (Figure 2). Other methods of synthesis have been described, such as phosphonalkylation [55,56], from bisphosphonate fractions [57,58], by a cross-coupling reaction [59], from aminomethylene-phosphonic acids [60,61], by radical reaction [62,63], from halo substrates [64], and from functional nitrogen substrates [65].

**Figure 3:**
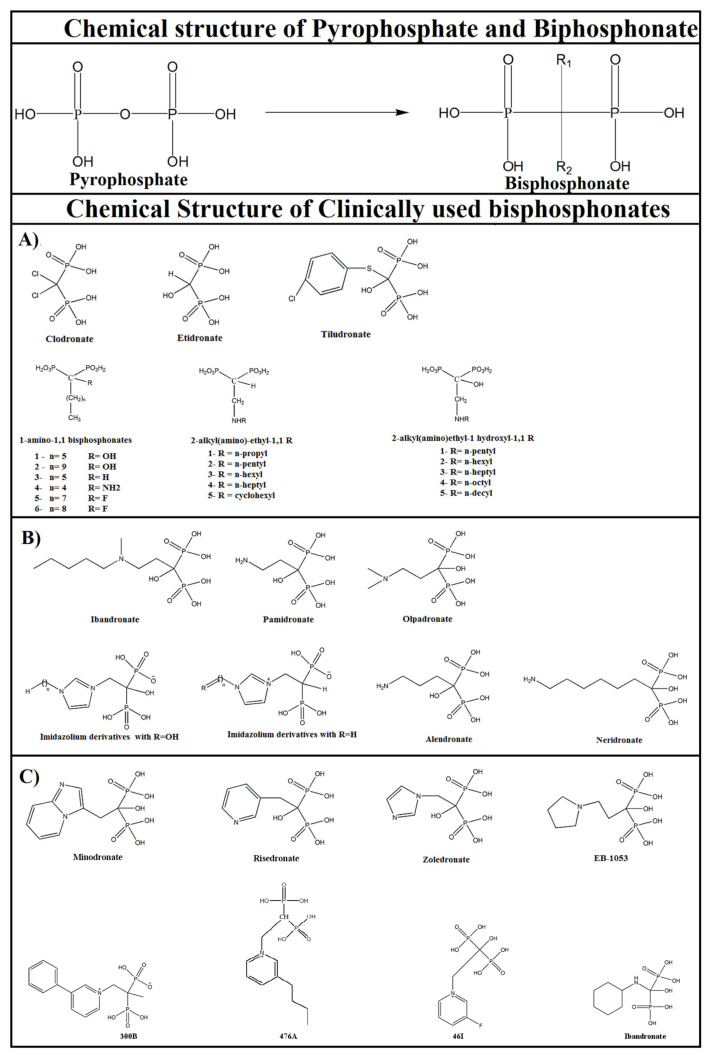
Chemical structures of pyrophosphonate, bisphosphonate, and bisphosphonates used clinically [41,85]. First generation (**A**); second generation (**B**); and third generation of bisphosphonates compounds (**C**).

Bisphosphonates are classified according to the presence or absence of nitrogen in their R2 side chain, as shown in Figure 3 [66]. Depending on the side chain inserted into the central carbon atom, the pharmacological characteristics of these compounds vary such as absorption, distribution, retention, elimination, renal excretion, and inhibition of cellular activity and mechanisms of action. Non-nitrogenated bisphosphonates, analogous to pyrophosphates, lead the cell to apoptosis owing to its toxicity when metabolized as adenosine triphosphate (ATP). However, nitrogenated bisphosphonates, similar to isoprenic pyrophosphate, are not metabolized and act by inhibiting the enzyme farnesyl pyrophosphate synthase (FPPS), thereby reducing prenylation of guanosine triphosphate (GTP)-binding proteins (such as Rho, Rab, Rac, and Cdc42) that are essential for osteoclast activity and survival [52,67,68].

Applications of bisphosphonates range from insecticides and herbicides [35,69] to drugs for the treatment of diseases such as osteoporosis, Paget’s disease, and parasitic diseases [27,70,71,72]. This comprehensive action is the result of the structural diversity of these compounds. The molecular variations of bisphosphonates over the decades allowed classifying them according to the synthesized structure. The first-generation bisphosphonates derivatives do not have nitrogen in their structures [73,74,75,76,77]. The second-generation compounds are of nitrogen-containing compounds with an alkyl chain of up to five carbon atoms [78,79]. Finally, third-generation compounds are compounds that have a branched N chain, or ring formation [80,81,82].

Compounds based on bisphosphonates belonging to the second and third generation are the main compounds with inhibitory activity on farnesyl pyrophosphate synthase (FPPS), an essential enzyme in the mevalonate pathway, responsible for the production of isoprenoids, such as cholesterol and ergosterol [83]. The mevalonate pathway plays a crucial biological role in eukaryotes. This metabolic pathway is responsible for the production of cholesterol/ergosterol, maintenance of cell membrane and organelles, biosynthesis of steroids, and other processes involving cellular signaling. Disorders in this metabolic pathway can cause toxicity, alteration of cellular structure and function, as well as loss of homeostasis [84].

Experimental evidence has shown that second- and third-generation nitrogenous bisphosphonates are generally more potent than conventional bisphosphonates in inhibiting bone resorption [14,82]. However, the main disadvantage of administering these compounds is related to toxicity, as it can induce the development of severe osteonecrosis and gastric inflammation in humans [70,86]. Even with toxicity, these nitrogen-containing compounds are still recommended in clinical practice, particularly in terminal cancer patients with hypercalcemia or bone metastasis, as these compounds act as calcium chelators. Furthermore, the use of these compounds is also justified in the process of bone resorption, preventing fractures and osteolytic progression, and consequently reducing bone pain [70].

**Figure 4:**
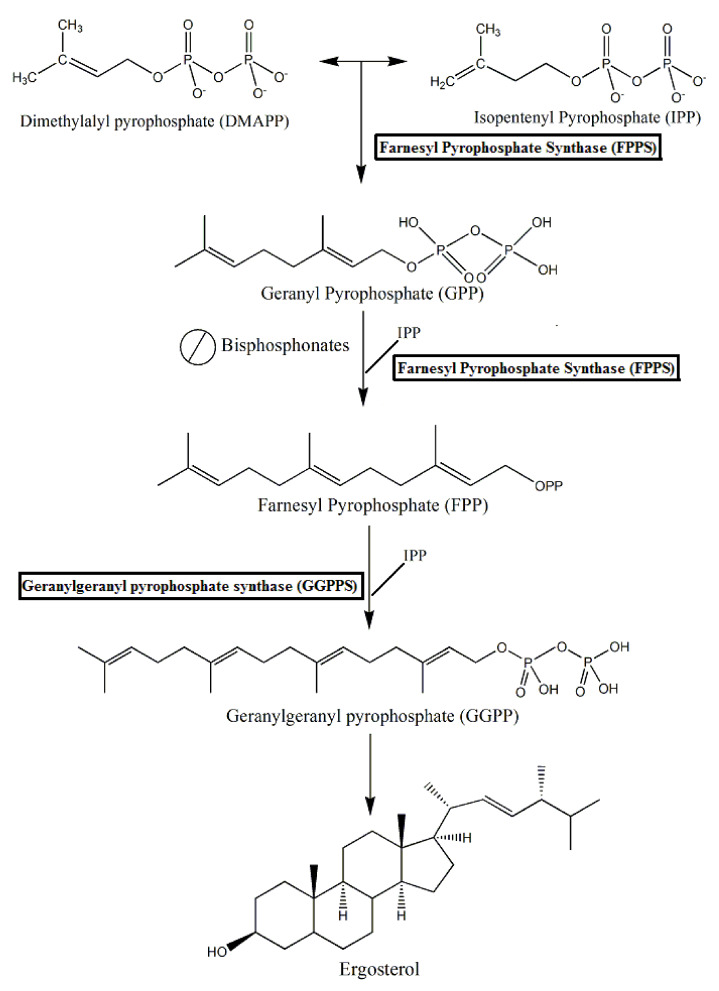
Schematic representation of the mevalonate pathway and the action of nitrogen-containing bisphosphonates. These compounds inhibit the enzyme farnesyl pyrophosphate synthase (FPPS), preventing the synthesis of farnesyl pyrophosphate (FPP) and geranylgeranyl pyrophosphate (GGPP) necessary for protein prenylation and subsequent ergosterol formation in eukaryotic cells.

Conventional bisphosphonates participate in metabolic reactions, mainly in ATP biosynthesis. On the other hand, nitrogenous bisphosphonates can act on the mevalonate pathway through the inhibition of the FPPS enzyme [14]. The FPPS enzyme catalyzes two-step reactions. In the first step, isopentenyl pyrophosphate (IPP) and its dimethylalyl pyrophosphate isomer (DMAPP) undergo condensation to form geranyl pyrophosphate (GPP) and farnesyl pyrophosphate (FPP), important isoprenoid intermediates [8,14]. Isopropenoids are essential molecules of the cellular machinery of eukaryotic and prokaryotic organisms, as an example, the trypanosomatids parasites and bacteria, respectively. These molecules are involved in several biological processes, such as cell differentiation and growth and antioxidant activity [40,87]. These intermediate precursors are essential for the formation of most isoprenoids, including ergosterol, a sterol whose inhibition results in changes in the integrity of the lipid bilayer of cells, leading to their death [8,88]. These reactions are represented schematically in Figure 4.

## 4. Bisphosphonate Compounds as Potential New Anti-*Trypanosoma cruzi* Drugs

American trypanosomiasis, also known as Chagas disease, was first described in 1909 by physician Carlos Chagas, after identifying and characterizing the etiologic agent, the hemoflagellate protozoan *Trypanosoma cruzi* [89]. Chagas disease is considered by the WHO to be an NTD that spreads beyond endemic countries through population migration. Among the countries affected are the United States, Canada, European countries, and some Pacific countries [90]. The main mechanisms of transmission of Chagas disease are vector, blood transfusion, organ transplantation, and congenital transmission, all of which contribute to the spread of the disease [90,91,92,93]. It is estimated that around 7 million people worldwide are infected with Chagas disease, with 6 million cases present in 21 countries in Latin America [94]. Furthermore, it is estimated that approximately 70 million people are at risk of contracting this disease, which is responsible for around 14,000 deaths per year [95]. Worldwide, 1% to 10% of children infected with *T. cruzi* are born with congenital Chagas disease [96] and the Pan American Health Organization (PAHO) estimates that 25% of new infections are congenitally transmitted [97,98]. Even in the USA, a non endemic country, the maternal-to infant transmission rate is estimated to be 1–5% [99]. *T. cruzi* infected infants may present with low birth weight, prematurity, hepatosplenomegaly, meningoencephalitis, and anemia [100,101,102].

Chagas disease can be considered as one of the most important NTDs in the world together with malaria and schistosomiasis. This disease presents a short period of acute phase—of eight to twelve weeks—and a prolonged chronic phase—of twenty to thirty years. In the chronic phase of the disease, the parasites may compromise the normal function of the cardiac apparatus, progressing to dilated cardiomyopathy that display cardiac arrhythmias, conduction abnormalities, and heart failure [103]. Other manifestations of this stage include digestive alterations, causing megacolon and megaesophagus, and consequently causing difficulties of swallowing, asphyxia, aspiration pneumonia, chronic constipation, and abdominal pain [94,96].

For more than 50 years, treatment for Chagas disease has been based on the use of two drugs, Benznidazole and Nifurtimox. Benznidazole is a nitroimidazole derivative, and its mechanism of action is supposed to be through reductive stress that involves covalent bonds to macromolecules vital to the parasite. Nifurtimox, in turn, is a nitrofuran that produces oxidative metabolites, such as oxygen peroxide, as the parasite does not have efficient mechanisms for detoxifying the produced substrates. In Brazil, Benznidazole is used as the drug of choice for the treatment of Chagas disease, despite its low effectiveness during the chronic phase of infection [92,104].

These drugs have pharmacological activity essentially in the acute phase of infection caused by *T. cruzi*, resulting in pharmacological efficacy of around 80% of treated patients. However, in the chronic phase of the infection, the effectiveness can vary from 7% to 10% for Nifurtimox and from 2% to 40% for Benznidazole [92]. In addition to the low pharmacological efficacy of these drugs in the chronic phase of infection, these compounds still have high toxicity, causing adverse reactions, such as anorexia, weight loss, neurological disorders (irritability, insomnia, disorientation, mood changes, paresthesia, and peripheral neuropathy), digestive manifestations such as nausea and vomiting, and sometimes fever and rash, leading to the patient often abandoning treatment [91,105].

Currently, Nifurtimox and Benznidazole are the two drugs used in the acute phase of the disease [106,107,108]. No effective treatment is available for the chronic phase of the disease. As the PAHO established in their 2018 recommendations, there is an urgent need to develop new drugs able to cure the trypanosome infection as well as drugs to reverse heart damage [98]. In this context, some enzymes involved in the synthesis of sterols in *T. cruzi* have been shown as potential targets for the development of anti-*T. cruzi* drugs. One of these enzymes is farnesyl pyrophosphate synthase (FPPS), which seems to be an adequate pharmacological target for the inhibitory action of bisphosphonates [32].

In *T. cruzi*, the use of bisphosphonates with antiparasitic activity seems to be feasible, as the R2 grouping of these molecules has the ability to interact and inhibit farnesyl pyrophosphate synthase (FPPS), causing a reduction in levels of FPPS and geranylgeranyl pyrophosphate (GGPP) [109]. The inhibitory action of these compounds on FPPS may play an important role in the functionality of these molecules by reducing the production of several sterols and poly-isoprenoids, such as farnesylated proteins [110,111], Heme-A [112], Dolichol-PP [113], ubiquinones [114], and the synthesis of ergosterol [115]. These molecules are of fundamental importance for the survival of the parasite, as they trigger relevant functions in the structure of membranes.

In this context, studies performed by Martin et al. [49] were the first to demonstrate that nitrogen-containing bisphosphonates inhibit the in vitro proliferation of *T. cruzi*, with IC_50_ (the concentration required to reduce the parasitemia by half) in the micromolar range. In addition, these compounds showed a broad range of antiparasitic activity in other trypanosomatids (*T. brucei* and *L. donovani*) as well as apicomplexan parasites (*T. gondii* and *P. falciparum*). In particular, zoledronate appeared to be the most effective, with an IC_50_ of 35 µM against *T. cruzi* amastigote replication. These studies also suggested that the observed effects of bisphosphonates were the result of the inhibition of sterol biosynthesis, at the level of FPPS enzyme [27].

Bouzahzah and collaborators [116] evaluated the antiparasitic activity of the risedronate compound in mice infected with *T. cruzi*. This study indicated that, when the compound was administered subcutaneously, there was a significant reduction in animal mortality. However, the myocardial phenomenon and the dilation of the animals’ right ventricle remained unchanged in the animals, when compared with the control group. According to Garzoni and collaborators [13], risedronate is capable of inducing antiparasitic activity by reducing the growth of *T. cruzi* epimastigotes in vitro. This antiparasitic phenomenon was characterized by the ability of this compound to deplete the parasite’s endogenous sterols. Thus, risedronate treated parasites showed a variety of changes such as ultrastructural changes including mitochondrial edema, disorganization of organelles such as reservosome and kinetoplast, along with the appearance of autophagic vesicles and progressive vacuolization of the cytoplasm. Additionally, risedronate also displayed antiparasitic activity in the amastigote form of *T. cruzi*, an intracellular form responsible for the chronic phase of Chagas disease.

Another interesting study by Montalvetti and collaborators reported that nitrogen-containing bisphosphonates—risedronate, alendronate, and pamidronate (IC_50_ ~7 nm–1 µM)—were more potent than non-nitrogen containing bisphosphonate and etidronate (IC_50_ ~58 µM) in inhibiting *T. cruzi* FPPS (TcFPPS) [22]. Similar results have also been reported for the human enzyme [117]. Pamidronate not only blocked the intracellular growth of *T. cruzi* amastigotes in vitro, but also substantially suppressed the proliferation of the parasite in vivo when tested in a murine model of acute Chagas disease [118]. It has been postulated that the antiparasitic activity of nitrogen-containing bisphosphonates could be owing to their preferential accumulation in the calcium and pyrophosphate-rich acidic organelles of the parasite named as acidocalcisomes [118]. A similar explanation has been suggested for the antiresorptive activity of these compounds that is based on their ability to bind avidly with calcium hydroxypatite in the bone mineral [29,119].

On the other hand, bisphosphonates derived from fatty acids, in which no nitrogen atom is present in R2 side chain, were also shown to be potent inhibitors of amastigote forms of *T. cruzi* as well as the target enzyme TcFPPS with IC_50_ values at the low micromolar level [120]. To further elaborate on non-nitrogenous bisphosphonates, there are at least three categories of these bisphosphonates derived from fatty acids that have been purposely designed as anti-parasitics [22,27,121]: 1-amino-1,1; 1-hydroxyl-1,1; and 1-alkyl-1,1 bisphosphonates (Figure 3A).

The inhibitory activity of 1-amino-1,1 bisphosphonates increases with length of the carbon chain [27]. For example, the compound with *n* = 4 inhibits TcFPPS with nanomolar affinity and is more potent than the 1-hydroxyl-1,1 bisphosphonate (*n* = 4) against TcFPPS [22]. However, 1-hydroxy class of bisphosphonates (*n* = 4 and 5) are potent inhibitors of TcFPPS compared with 1-alkyl bisphosphonates (*n* = 4 and 5). On the other hand, the fatty acid fluorine derivatives of bisphosphonates have relatively lower activity against *T. cruzi* amastigotes, but are potent inhibitors of *T. gondii* tachyzoites, with IC_50_ in the low nanomolar range [122].

The differential biological activities of different bisphosphonate pharmacophores across trypanosomatids are further displayed in the 2-alkyl(amino)-ethyl-1,1 bisphosphonate series (Figure 3A). The 2-alkyl(amino)-ethyl derivatives (*n* = 5 and 6) have higher biological activity than their counterparts 2-alkylaminoethyl-1-hydroxy-1,1 bisphosphonates against *T. cruzi* amastigotes [22,50,121,123]. Furthermore, the 2-alkyl(amino)ethyl derivatives with *n* > 3 are more potent (IC_50_ in the nanomolar range) than the parent pharmacophores, 1-hydroxy, 1-alkyl, and 1-amino bisphosphonates, as growth inhibitors of trypanosomatids. Surprisingly, the 2-alkylaminoethyl-1-fluoro-1,1 bisphosphonates synthesized by Galaka et al. by replacing hydrogen with fluorine atom at C-1 position rendered these compounds inactive for *T. cruzi* amastigotes as well as *T. gondii* tachyzoites [40].

However, it is important to note that most clinically approved drugs for bone-related disorders are nitrogen containing owing to their superior ability in suppressing osteoclast survival. These constitute the second and third generation of bisphosphonates with nitrogen containing R2 side chain. Thus, some novel strategies have been reported to increase the potency of these nitrogen containing bisphosphonates for repurposing as antiparasitic drugs. In one such study, bioactive ligand risedronate was coordinated with different first row transition metal ions—Cu, Co, Mn, and Ni—in an effort to generate a synergistic and additive effect of the ligand metal complex. These complexes in particular ‘Mn risedronate complex’ demonstrated stronger inhibition (IC_50_ ~14 µM) with low cytotoxicity in mammalian Vero cells compared with the free risedronate ligand (IC_50_ ~55 µM) against proliferation of *T. cruzi* amastigotes [124]. Similarly, bisphosphonate ibandronate was also complexed with these transition metals and evaluated for anti-proliferative effects on TcFPPS. In addition to exhibiting improved inhibition of *T. cruzi* amastigotes, these metal complexes also showed a poor inhibition for human FPPS (IC_50_ ~>10 µM) than free ibandronate (IC_50_ ~0.96 µM) [121].

In another study by Yang and colleagues, bisphosphonates for treatment of trypanosomiasis were selected on the basis of the enzymatic activity inhibition as well as cell growth inhibition with low human cell toxicity as assessed in HEK293 cells. In this study, the zoledronate derivatives containing imidazolium side chain with *n* = 0–8, with and without 1-hydoxyl (1-OH) in the bisphosphonate backbone, were as potent as bisphosphonates with pyridinium side chain against TbFPPS (Figure 3B) [83]. Thus, the antiparasitic activity of bisphosphonate compounds against *T. cruzi* parasites in vitro as well as in vivo justifies the need for further research that can contribute to the development of these compounds as potential new drugs for the treatment of Chagas disease.

## 5. Bisphosphonate Compounds as Potential New Anti-*Leishmania* spp. Drugs

Leishmaniasis is a group of parasitic diseases caused by trypanosomatids belonging to the genus *Leishmania* [125]. These trypanosomatids affect humans; several species of wild and domestic mammals; as well as invertebrates belonging to the order Diptera, family Psychodidae, genus *Lutzomya* in the New World, and genus *Phlebotomus* in the Old World [126]. The *Leishmania* specie is classified into two subgenera—*Leishmania Viannia* and *Leishmania Leishmania*—according to their clinical and epidemiological characteristics [127].

According to WHO [128], leishmaniasis is endemic in tropical and subtropical areas and in the Mediterranean basin, affecting 98 countries and about 12 million people. It is estimated that more than 1 billion people live in endemic regions at risk of infection. In addition, about 1.3 million new cases of the disease are registered annually, and mortality is 20,000 to 30,000 per year [128,129]. This disease is endemic in five continents, in 98 countries located mainly in tropical and subtropical regions, where socioeconomic status impacts disease control [95,96,97]. The clinical forms of leishmaniasis are visceral leishmaniasis (VL) and cutaneous leishmaniasis (CL) [130]. The factors that determine the different clinical forms are the species of the infectious agent, which vary according to the geographical area.

In the last decades, few drugs have been made available for the treatment of leishmaniasis, among which none is suitable for treatment owing to high toxicity, prohibitive prices, intravenous administration that requires hospitalization [131], and the risk of developing drug resistance [118]. In Brazil, mainly in northeastern Brazil, leishmaniasis presents a variety of clinical spectra, characterized by tegumentar leishmaniasis and visceral leishmaniasis. Owing to the exposure of U.S. troops to both visceral and cutaneous leishmaniasis, after the deployments in the Middle East, the United States has observed infections. In fact, the army has developed a topical to treat cutaneous leishmaniasis. As of 2007, about 1300 soldiers have been diagnosed with leishmaniasis [132,133,134], but the real problem is the threat of leishmaniasis infection of autochthonous cases within the USA and the expansion of the natural borders of *Leishmania mexicana* beyond Texas [135,136,137].

The drugs approved by the FDA for the treatment of leishmaniasis are as follows: (i) Pentavalent antimonials—meglumine antimoniate and sodium stibogluconate—first-line drugs of choice, but when accumulated in organic tissues, they can cause serious adverse effects, such as vomiting, severe nausea, anorexia, headache, arthralgia, lethargy, cardiotoxicity, pancreatitis, and nephrotoxicity [138]; (ii) Amphotericin B, also of the first line of choice; however, it can cause chills and fever, associated with myocarditis and nephrotoxicity, in addition to a high cost for liposomal formulation [139]; (iii) Pentamidine, a second-line drug for patients resistant to antimony; however, it was discontinued owing to severe adverse reactions such as myocarditis, headache, hypotension, hyperglycemia, and hypoglycemia [140]; (iv) Paromomycin, an aminoglycoside antibiotic used in the topical treatment of CL and as an alternative to VL; however, it causes respiratory dysfunction and changes in lipid metabolism [117]; and (v) Miltefosine, an oral therapeutic drug for the treatment of all forms of leishmaniasis, which was approved in 2014 [141,142]. However, the use of Miltefosine is contraindicated for pregnant women and nursing mothers, because it is a teratogenesis drug.

Besides the limitations of currently used drugs, the failure of treatment owing to low plasma concentration [143] highlight the necessity to develop effective anti-leishmaniasis drugs with reduced side effects. In recent years, several approaches have been reported to identify and optimize bisphosphonates as new drugs against *Leishmania* spp. parasites [144]. Initial studies by Martin at al. evaluating the activities of various nitrogen containing bisphosphonates on the intracellular proliferation of L. donovani amastigotes demonstrated that risedronate was most effective with an IC_50_ of 2.3 μM with little toxicity to host cells [27]. Further studies on nitrogen-containing bisphosphonates as potential anti-Leishmania drugs were performed by Yardley and collaborators [25]. These authors carried out preclinical tests in mice infected with *L. donovani* and evaluated the antiparasitic activity in vivo of three bisphosphonate-based compounds, namely, alendronate, pamidronate, and risedronate. Alendronate did not show antiparasitic activity against *L. donovani*. However, the compounds pamidronate and risedronate showed antiparasitic activity when administered by intravenous or intraperitoneal routes. In 2002, another study published by Rodriguez et al. demonstrated the antiparasitic activity of pamidronate in an experimental model of *L. mexicana amazonensis* [10]. Pamidronate, when administered intraperitoneally with a dose of 10 mg/kg/day, for five days, cured mice with cutaneous leishmaniasis with a healing process characterized by the extinction of lesions over time and the disappearance of amastigote forms at the lesion site, as determined by polymerase-chain reaction.

To investigate the target of bisphosphonates, Ortiz-Gómez and collaborators studied the effects of bisphosphonate risedronate on *L. major* parasites overexpressing FPPS. In these studies, *L. major* promastigotes were transfected with FPPS and the resulting tranfectants were characterized for the overexpression of FPPS. The IC_50_ for risedronate against these parasites overexpressing LmFPPS was found to be 70 times higher than the wild-type cells, indicating the correlation of degree of resistance with the increase in enzyme activity. Furthermore, when resistance was induced by stepwise selection with increasing concentrations of risedronate, the resulting resistant promastigotes exhibited a fourfold increase in the levels of FPPS as a result of drug pressure. These studies further suggested that FPPS is the main target of amino bisphosphonates in *Leishmania* [9].

Another interesting study by Gadelha and colleagues evaluated the effects of N-BPs on the cell viability and ultrastructure of *L. infantum*, the FPPS of which is about 97% identical to that of *L. major*. The N-BP risedronate had stronger anti-proliferative activity against *L. infantum* promastigotes with an IC_50_ of 13.8 μM compared with other N-BPs such as ibandronate (IC_50_ = 85.1 μM) and alendronate (IC_50_ = 112.2 μM). In addition, the three N-BPS displayed the same ultra-structural alterations in *L. infantum* promastigotes, such as accumulation of small vesicles in the Golgi region, mitochondrial swelling, altered cell division, formation of intracellular vesicles and lamellae, plasma membrane blebbing, as well as nuclear pyknosis and chromatin condensation [145].

To further understand the interaction of N-BPs with the target enzyme FPPS in *L. major*, the binding affinities of nitrogen-containing bisphosphonates derivatives with pyridinium (e.g., 300B, 46I, and 476A) or imidazolium side chains (e.g., zoledronate/91B) (Figure 3C) were probed by isothermal titration calorimetry. The binding affinity or K_d_ values for LmFPPS against these inhibitors were in the range of 28–343 nM. Of particular interest was zoledronate, as it exhibited five times higher affinity (K_d_ ~28 nM) for *Leishmania major* FPPS compared to human FPPS (K_d_ ~150 nM) [7,146]. Furthermore, zoledronate was also found to have an inhibition constant or Ki of 11 nM for LmFPPS using inhibition studies, suggesting its high potency [123]. The differential activities and affinities of these bisphosphonates for human and *Leishmania* FPPS enzymes could be greatly exploited to design more effective parasite-specific inhibitors. Despite these initial studies on the use of bisphosphonates in experimental leishmaniasis therapy, further in vivo studies are needed to validate the use of these compounds as potential parasitic-specific therapies for the treatment of leishmaniasis.

## 6. Overall Structure of LmFPPS and TcFPPS in Complex with Inhibitors

**Figure 5:**
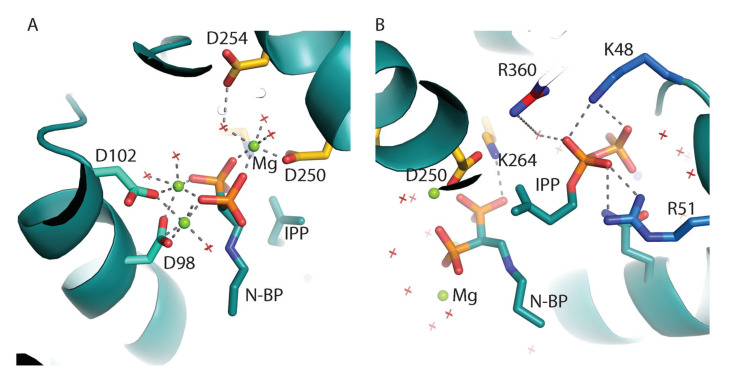
Active site of *Trypanosoma cruzi* farnesyl pyrophosphate synthase (FPPS) in complex with a N-BP ([2-(n-propylamino)ethane-1,1-diyl] bisphosphonic acid), Mg^2+^, and isopentenyl pyrophosphate (IPP) (PDB ID 4DXJ). (**A**) Coordination of Mg ^2+^ is shown with grey dashed lines. Residues of the aspartate rich motifs are shown as sticks. Water molecules are shown as red crossmarks. (**B**) IPP is at hydrogen bonding distance of K48, R51, and R360.

FPPS enzymes are dimers with an extensive buried surface area of about 3000 Å^2^ [147]. The structures of trypanosomatids and human FPPS in complex with a bisphosphonate inhibitor (risedronate/zoledronate or other N-BPs), substrate IPP, and three divalent cations (either Mg^2+^ or Ca^2+^) have been determined by X-ray crystallography [7,11,147,148] (Figure 5A). In the structures of these complexes, the nitrogen containing BP inhibitors occupy the allylic site and IPP (when present) occupies the homoallylic site. An overall ‘closing’ of the active site is observed in the structures of these complexes when compared with the apo structure [148]. Three divalent cations bridge the side chains of aspartate residues from the two aspartate-rich motifs (DDXXD; residues 98–102 in the first aspartate-rich motif and residues 250–254 in the second aspartate-rich motif) to coordinate the bisphosphonate atoms of the inhibitor bound at the allylic site [83,147,148]. Each divalent cation is coordinated by water molecules and oxygen atoms of the bisphosphonate. On the other hand, the phosphate oxygen atoms of the IPP are recognized by positively charged residues (K48, R51, and R360) without the involvement of divalent cations (Figure 5B).

## 7. Conclusions and Perspectives

NTDs represent a group of diseases that affect millions of individuals worldwide, resulting from the social and economic underdevelopment of developing countries [2]. In the context of these diseases, those caused by parasitic protozoa, such as Chagas disease and leishmaniasis, deserve special attention, mainly owing to the few therapeutic options, high toxicity associated with currently used drugs, difficulty of access, high cost, and low efficacy that interfere with treatment adherence. Thus, there is an urgent need to develop new drugs with novel modes of action that are more effective, cheaper, less toxic, and easy to administer.

In this sense, bisphosphonate compounds are presented in this work as potential new compounds with antiparasitic activity, as these compounds can be easily synthesized in different ways, facilitating the rational design of new molecules with high antiparasitic activity and low toxicity. On the other hand, some of these compounds are already being used in pharmacological therapy for bone diseases in humans, thus facilitating the practice of repositioning drugs.

Thanks to the ability of some of these compounds to inhibit the mevalonate pathway in different types of protozoa, mainly owing to their ability to inhibit the enzyme FPPS, an enzyme essential for isoprenoid biosynthesis in trypanosomatids, nitrogenous bisphosphonates appear to be promising for the study of structure-activity mechanisms of new compounds with antiparasitic activity. However, in addition to the pharmacological action, knowledge of the synthesis process of these compounds can facilitate the development of new ones that are less toxic and can be used more safely in the context of pharmacological treatment of Chagas disease and leishmaniasis. In conclusion, further pharmacological studies, chemical synthesis, and toxicology are necessary to move these compounds forward for use in antiparasitic drug therapy.
